# Large gastric trichobezoar causing failure to thrive and iron deficiency anaemia in an adolescent girl: a case report emphasising the imaging findings and review of the literature

**DOI:** 10.1259/bjrcr.20180080

**Published:** 2019-01-11

**Authors:** Duncan Lyons

**Affiliations:** 1 Launceston General Hospital, Launceston, TAS, Australia

## Abstract

Failure to thrive, iron deficiency anaemia and abdominal pain are common paediatric presentations to general practitioners, outpatient clinics and are often referred to emergency departments. When young female patients suffering from psychiatric disorders, such as trichotillomania and trichophagia present to medical practitioners, the rare diagnosis of a trichobezoar, which is an accumulation of indigestible human hair in the gastrointestinal tract (90 % occurring in the stomach) needs to be suspected. Imaging is the mainstay of trichobezoar diagnosis and requires accurate interpretation to prevent complications. A case of a 14-year-old girl is presented, who was referred from paediatric outpatient clinics for an elective admission to the emergency department. She presented with abdominal pain, iron deficiency anaemia, failure to thrive and an epigastric/left upper quadrant mass felt on examination. A large trichobezoar was found on CT images, confirmed on endoscopy and removed with an open laparotomy. However, on the work-up imaging modalities, the radiologists missed the subtle findings of a trichobezoar. Although uncommon, trichobezoars should be considered as a differential diagnosis in female paediatric patients with a psychiatric history, who present with abdominal pain and epigastric mass. Imaging is the mainstay for trichobezoar diagnosis. As such, radiologists need to be familiar with the apparent, and subtler, pathological findings of this diagnosis and possible differential diagnoses across all imaging modalities. After successful treatment, psychiatric consultation and treatment is imperative in order to prevent reoccurrence.

## Introduction

Bezoars are rare collections of hard non-digestible foreign bodies that usually accumulate in the stomach and/or other areas of the gastrointestinal tract.^1^ There are several types of bezoars, which are classified depending on their primary constitute. The most common ones are phytobezoar (vegetable), trichobezoar (hair), and lactobezoar (milk formula).^2^ The majority of patients presenting with trichobezoar formation are young females who suffer from psychiatric disorders, such as trichotillomania (compulsive hair pulling) and trichophagia (hair ingestion).^3^ Patients with a trichobezoar may be asymptomatic, which can result in late presentation and, when left chronically undiagnosed, it can lead to severe anaemia through either gastrointestinal bleeding or malabsorption.^4^ This can result in failure to thrive.^4^ Imaging is the mainstay of diagnosis and, therefore, both clinicians and radiologists need to have a high level of suspicion in order to diagnose the condition early and prevent serious complications. This report describes a chronic trichobezoar in an adolescent female and highlights the features of diagnosis, in addition to the apparent and subtler imaging findings that may be missed by radiologists.

## Case Presentation

A 14-year-old female was referred by her paediatrician in outpatient clinic on 30 January 2018 for an elective hospital admission on 1 February, 2018. She was referred because she had recently lost weight; was complaining of a 2-week history of intermittent epigastric abdominal pain; and, upon investigation, the paediatrician found that she had an iron deficiency anaemia and an epigastric/left upper quadrant mass was found on clinical examination. She had lost 1 kg of weight in the past week, but had a normal appetite. The abdominal pain was not associated with nausea, vomiting, diarrhoea or other constitutional symptoms. She had noticed some dark stools, (consistent with her being on Ferrograd C) and a slight reduction in her exercise tolerance, but felt well within herself.

She had been seen in the paediatric outpatient clinic due to her history of congenital adrenal hyperplasia. Other relevant past history includes delayed puberty and poor growth, with poor weight and height gain in the past 3 years. Further history revealed that she had been suffering from the abdominal mass and abdominal pain for approximately a year, having had roughly 3–4 episodes during this time. An abdominal ultrasound was ordered in June 2017 to investigate, which showed no abnormalities, apart from a small left kidney. The girl’s medications included Hydrocortisone, Fluticasone and Ferrograd C. Her mother provided consent for medical imaging and procedures.

On admission, physical examination revealed a firm and non-tender mass in the epigastric and left upper quadrant region. Her abdomen was mildly distended, but soft and non-tender. Examinations of her heart and lungs revealed no abnormalities. Additionally, there was no evidence of mouth ulcers, skin changes, obvious alopecia or eye irritation. Her weight was 34.6 kg and height 146 cm (first percentile). Her vital signs showed no significant abnormalities. The differential diagnosis at this stage was thought to be of spleen pathology. The plan on admission was for laboratory investigations, stool tests, commence an iron infusion, an abdominal ultrasound and chest radiograph. On admission (1 February 2018) her abnormal blood tests showed: Hb 76 g l^−1^ (L), MCV 67fL (L), Albumin 32 g l^−1^ (L), Ferritin <5 ug l^−1^ (L) and ESR 25 mm hr^–1^ (H). Extensive urine, stool and blood tests (including autoimmune, endocrine, infective and vitamin testing) were all negative.

**Figure 1.**
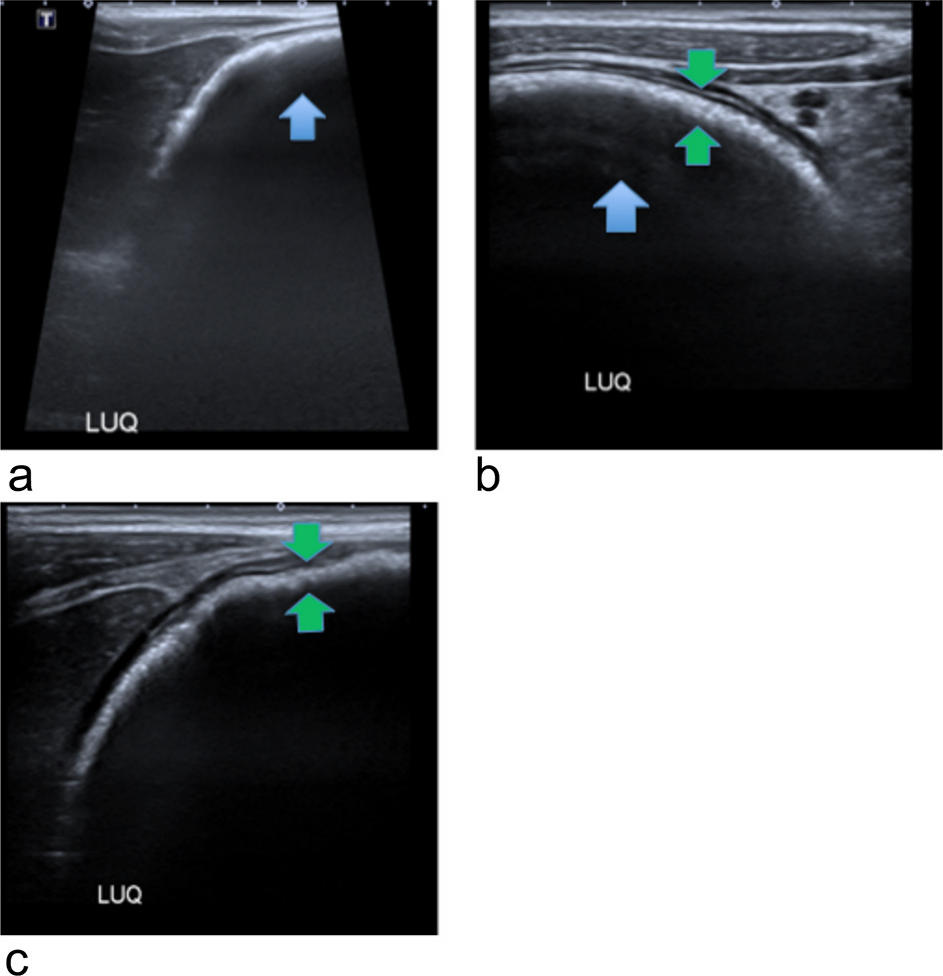
Abdominal ultrasound on 1 February 2018: demonstrating an echogenic curved band (green arrows) with posterior acoustic shadowing (blue arrows) in the left upper quadrant of the abdomen. LUQ, left upper quadrant.

**Figure 2.**
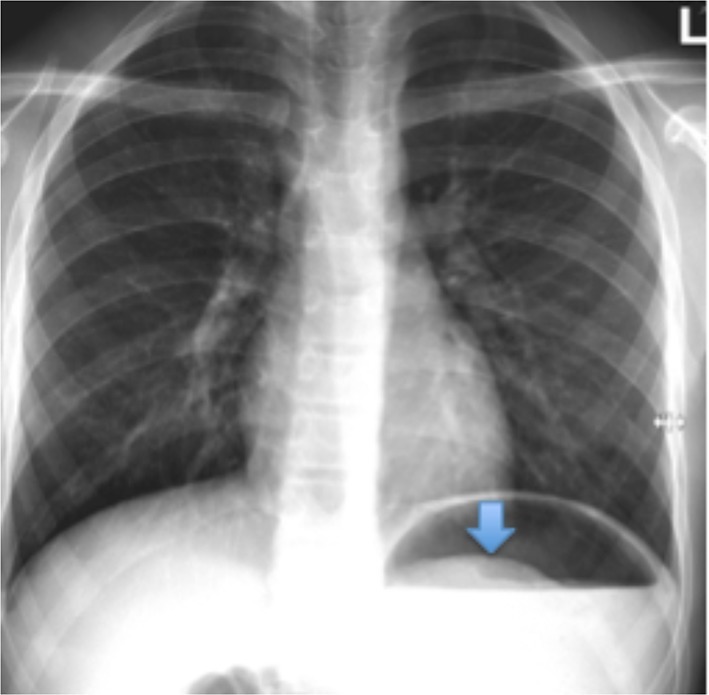
CXR on1 February 2018: demonstrating the upper portion of the large bezoar projecting into the gastric air bubble (blue arrow), with no signs of pneumoperitoneum or other complications.

**Figure 3.**
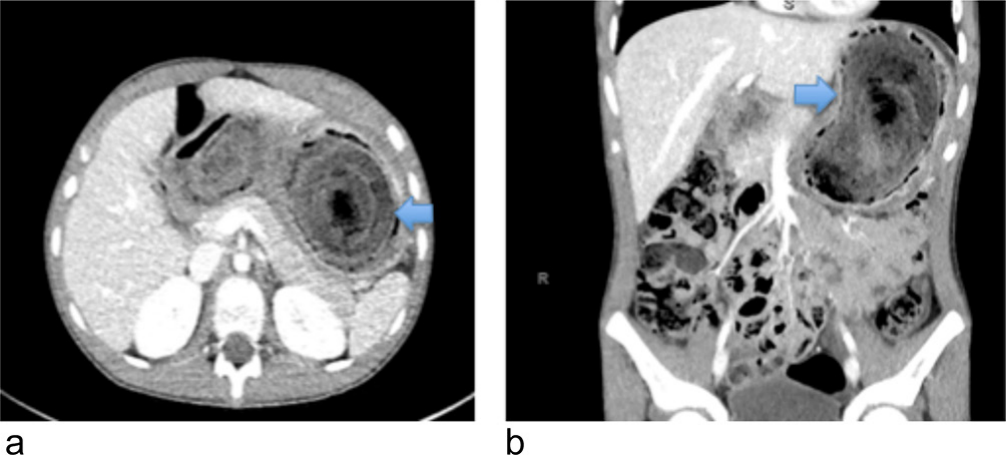
Contrast enhanced axial and coronal CT C/A/P on 1 February 2018: demonstrating a huge oval, heterogeneous, well defined, solid appearing, non-enhancing intraluminal mass, measuring 13 × 6 × 6 cm, in a distended stomach (blue arrows). With no extension of the large mass through the pyloric canal.

Abdominal ultrasound on the 1 February 2018 showed grossly enlarged lymph nodes at the left upper quadrant, free intraperitoneal fluid at the right iliac fossa and an atrophic left kidney with cystic changes in the lower pole. The paediatric team requested a second read, which found a prominent bowel with the cause of the findings unclear. The report conclusion stated that these findings were atypical for mesenteric adenitis and CT or MRI would be appropriate. However, on ultrasound, both radiologists missed the finding of a large thick and curved echogenic band with posterior acoustic shadowing (Figure 1a–c), which is typical of a trichobezoar. This diagnosis needs to be differentiated from other differentials including food remains, barium concretions (if used) and bile ileus. Given the initial abdominal ultrasound findings, a chest radiograph was requested to check for mediastinal lymphadenopathy. The chest radiograph was reported by the radiologist as having no evidence of mediastinal lymphadenopathy or any other pathology. However, the radiologist missed the subtle evidence of a trichobezoar on plain chest radiography. It is seen as a curvilinear shadow below (Figure 2), and projecting into the gastric air bubble. This finding could easily have been confused for normal food contents. Later that evening, a CT scan of the chest, abdomen and pelvis with i.v. contrast was performed (Figure 3a,b). This showed a grossly distended stomach filled by a suspected solid content, surrounded by a rim of gas, occupying as much as 13 × 6 × 6 cm in size. Additionally, mild to moderate free fluid and adjacent perigastric nodes anteriorly were found with no obvious duodenal lesion found. The suspected diagnosis was a gastric bezoar. The following day the patient admitted to pulling/playing with her hair and eating a substantial amount of hair as a child, but denied having consumed any for years. The gastroenterology team were consulted and an upper gastrointestinal endoscopy was organised later that day. This showed a large gastric trichobezoar with an associated gastric ulcer on the lesser curve of the stomach. The duodenum appeared unremarkable to the D2 segment. The general surgical team were subsequently consulted and she underwent a laparotomy, gastrotomy and retrieval of the trichobezoar on the 3 February 2018.

She recovered uneventfully post-operatively, being discharged from hospital after 4 days. Her last bloods before discharge were on 5 February 2018, which showed a Hb of 86 (L), MCV 73 (L), Alb 26 (L). She was discharged with nil issues from the general surgical outpatient clinic after her first visit 3 weeks post-discharge from hospital. On her most recent paediatric outpatient appointment on the 17 April 2018, she had been very well, with increased energy, exercise tolerance and her blood results had normalised.

## Discussion:

Trichobezoars are typically found in the stomach, however in approximately 10% of cases they can extend into the small intestine, even the colon, and this condition is labelled Rapunzel syndrome.^5^ 90% of trichobezoars occur in females, with 80% of these occurring in females under the age of 30—primarily children and adolescent females.^6,7^ Predisposing factors of trichobezoars include psychiatric disorders, such as trichotillomania, trichophagia, depression, anxiety and poor self-image.^8,9^ Trichotillomania has a bimodal age of onset, between the ages of 7 and 8 or in early adolescence, between the ages of 11 and 12.^10^ Approximately 1 in 2000 children in the world suffer from trichotillomania and approximately 30% of these children will also suffer from trichophagia. Of trichophagia sufferers, only 1% will develop a trichobezoar.^11^ Although trichobezoar is usually associated with psychiatric disease, there is evidence that the condition may affect healthy females.^12^

Human hair is indigestible in the gastrointestinal tract because of its smooth and slippery surface and its enzyme-resistant properties.^13^ The hair becomes stagnant and accumulates between the gastric mucosal folds.^9^ With continued hair ingestion, this leads to impaction with food, air and mucus, resulting in trichobezoar formation.^9,13^ Patients with trichobezoar may be asymptomatic for years. Symptoms develop as the trichobezoar enlarges and causes obstruction.^6^ Common symptoms of trichobezoars include: epigastric discomfort, abdominal pain, nausea and vomiting, weight loss, asthenia and constipation (more commonly obstruction) or diarrhoea.^14^ Other symptoms can include anorexia, pain related to food consumption, halitosis or peritonitis.^9^ Symptoms of iron deficiency anaemia and malabsorption may also be present, due to the complications of trichobezoars.^15^ On clinical examination a palpable abdominal mass is found in over 80% of patients, and other findings may include halitosis and alopecia.^14^ If a trichobezoar is left undetected or untreated, it can lead to harmful complications including: failure to thrive, gastric ulceration and bleeding, gastric or duodenal perforation, upper gastrointestinal haemorrhage, peritonitis, pancreatitis, iron deficiency anaemia, malabsorption, intussusception, biliary perforation, jaundice and fistula formation.^6,16–19^

Diagnosis of a trichobezoar utilises imaging and often direct visualisation through upper gastrointestinal endoscopy. Abdominal radiographs can be used to aid the diagnosis of a gastric trichobezoar. The conventional findings are a mottled soft-tissue opacity filling the stomach that is often distended and may show a calcified rim.^20–22^ However, during the literature review for this case report, there was no research that supported chest radiography to aid in the diagnosis of a trichobezoar. Instead, chest radiography is typically used to detect complications, such as a pneumoperitoneum. Upper gastrointestinal fluoroscopic imaging can show a gastric or duodenal mottled intraluminal filling defect.^5,20^ Further filling defects within the bowel are likely due to the bezoar breaking down.^22^ Ultrasound is often used as first-line imaging, however it has a very low sensitivity in diagnosing gastric bezoars. This is because hair has a high echogenicity, trapped air bubbles and various acoustic reflections.^21–23^ When positive, ultrasound can demonstrate an echogenic curved band with posterior acoustic shadowing.^21,23^ Abdominal CT is the preferred modality for the diagnosis of a trichobezoar, with the diagnostic accuracy identified to be between 73 and 95%.^24,25^ CT demonstrates a well-demarcated, mottled oval intraluminal mass.^20–22^ The mottled appearance of a trichobezoar demonstrated on CT is due to the hair mixed with trapped air and ingested food.^26^ It can be difficult to distinguish retained food from a bezoar, however a bezoar is usually oval/round and fills the gastric lumen with diffuse air bubbles throughout the mass.^21^ Additionally, the serious complications detailed in the above paragraph, can all be demonstrated on CT.^21,22,24^ For the diagnosis work-up of a trichobezoar, it has been demonstrated that there is no advantage of MRI over CT.^17,20^ Additionally, CT is more readily available, less time consuming, cheaper and on MRI, bezoars can easily be confused with air due to their signal appearances.^20^ Used in conjunction with imaging, upper gastrointestinal endoscopy allows direct visualisation of the bezoar and it can help determine its extent, as well as allowing sampling of the mass and if possible, therapeutic intervention.^26,27^

Treatment of a trichobezoar includes treating the physical bezoar and managing any underlying psychiatric concerns. Traditionally, a gastric trichobezoar was treated with laparotomy with either gastrotomy or enterotomy and this is still the typical treatment for large trichobezoars and Rapunzel Syndrome.^6^ Small to moderate-sized trichobezoars can be removed by laparoscopic techniques.^17^ Endoscopic approaches use a range of devices to cause fragmentation of the trichobezoar, they include: forceps, snares, bezotomes, bezotriptors, needle-knife and plasma coagulation with argon.^15^ A study found that only 5% of attempted endoscopic removals of trichobezoars were successful, whereas 75% of laparoscopic surgical extractions were successful and 99% of all laparotomy followed by gastrotomy cases were successful.^13^ Although there have been advances in pharmacological treatment for other types of bezoars, it is normally ineffective in trichobezoars.^12,28^ The high failure rates have led to non-operative treatments for trichobezoars being discouraged. In order to prevent reoccurrence, treatment of the underlying psychiatric disorder is required. Behavioural therapy (primarily cognitive behavioural therapy) for the treatment of trichotillomania and trichophagia has been shown to provide patients with an excellent long-term prognosis.^15,20,29^ Cognitive behavioural therapy additionally helps with any underlying depression/anxiety that the patient may be experiencing.^29^ Long-term psychiatric follow-up is heavily recommended.^15,20,29^

## Learning points:

- Although rare, trichobezoars should be considered as a differential diagnosis in young female paediatric patients (particularly those with a psychiatric history or history of trichotillomania or trichophagia), who present with an iron deficiency anaemia, failure to thrive and an epigastric/left upper quadrant mass.
- Imaging is the mainstay for trichobezoar diagnosis. As such, radiologists need to be familiar with both the apparent, and subtler, pathological findings of this diagnosis and possible differential diagnoses across all imaging modalities.
- This is particularly important in paediatric and young adult patients, where the use of CT scans (where the findings of a trichobezoar are more apparent) is often limited due to the ionizing radiation, potentially delaying diagnosis.
- An accurate and timely diagnosis can improve clinical management and reduce morbidity and mortality associated with trichobezoars.
- Gastric trichobezoars are best treated through the collective minds of multidisciplinary teams.
- After successful treatment, psychiatric consultation and treatment is imperative in order to prevent reoccurrence and improve long-term prognosis.
